# Availability and use of web-based interventions for patients with head and neck cancer: a scoping review

**DOI:** 10.1007/s11764-022-01168-1

**Published:** 2022-01-28

**Authors:** Rosemary Kelly, Peter Gordon, Ruth Thompson, Cherith Semple

**Affiliations:** 1 School of Nursing, Ulster University, Shore Road, Newtownabbey, Co Antrim BT37 0QB Ireland; 2 South Eastern Health and Social Care Trust, Cancer Services, Ulster Hospital, Upper Newtownards Road, Belfast, BT16 1RH Ireland

**Keywords:** Head and neck cancer, Scoping review, Web-based interventions, Online resources, Patient preferences, Shared decision-making

## Abstract

**Purpose:**

To identify and review the nature, scope and use of web-based interventions for patients with head and neck cancer (HNC).

**Method:**

A scoping review guided by the methodological framework described by the Joanna Briggs Institute was performed to review empirical studies and websites. Seven electronic databases (CINAHL, Medline, Scopus, Embase, Cochrane, PubMed and PsycInfo) were searched from 2010 to 2020, data extracted and synthesised using thematic analysis. The Google search engine was employed, identifying the first 100 websites, using the search term *head and neck cancer.* Websites meeting eligibility criteria were assessed using the QUEST analysis tool, and descriptively summarised.

**Results:**

Thirteen empirical studies and 32 websites were included. As identified by empirical studies, web-based interventions were developed to provide (1) patient information on HNC and related treatments, (2) advice and support during treatment and (3) management strategies promoting adjustment to life with and beyond HNC. The reviewed websites provided minimal information to aid shared decision-making and facilitate preparedness for treatment, with few utilising patient narratives. Web-based interventions for HNC patients were mainly text based and focused on survivorship.

**Conclusions:**

There is a paucity of theory-based, co-designed web-based interventions using patient narratives.

**Implications for Cancer Survivors:**

As patients increasingly look to the internet for advice and support, healthcare professionals are in a position to provide high-quality web-based interventions. There is an opportunity to rigorously develop a web-based intervention, containing narratives of peoples’ lives before and after HNC treatment, aiding decision-making, preparedness for treatment and self-management.

**Supplementary Information:**

The online version contains supplementary material available at 10.1007/s11764-022-01168-1.

## Introduction

Globally, head and neck cancer (HNC) affects approximately 550,000 people annually [1]. This represents a wide-ranging group of cancers arising from the epithelial lining of the upper aerodigestive tract, and affecting the oral cavity including the lips; pharynx; larynx; paranasal sinuses and nasal cavity; salivary glands and middle ear [2]. The incidence of HNC is increasing in the United Kingdom (UK) and aetiology and demographics of patients are changing; for example, HNC is affecting younger people from more affluent backgrounds, due to increasing HPV-related disease [3]. The incidence rates in the UK have risen by a third in the last three decades [4], with treatments and outcomes varying according to tumour histology, site and stage. Current treatment modalities for HNC with curative intent are surgery, radiotherapy and chemotherapy, or a combination approach [5]. The impact of treatment on function (speech and swallow) and appearance varies hugely, depending on the HN subsite and treatment modalities, and consequently affects individual’s social, work and personal relationships [6–8]. Clinical teams can face ethical, emotional and practical challenges when endeavouring to convey realistic and relevant views on the side effects and outcomes from the different treatment modalities available for the management of HNC [9]. Provision of this information is paramount, to promote shared decision-making [10].

Prior to the COVID-19 pandemic, HNC survival rates were slowly improving; hence, more people were living with the effects of their cancer and its treatment to include physical and psychosocial disruption and a diminished sense of self [6]. In an era with improved long-term survival rates, it is increasingly important that patients receive person-centred care, to include stratified information relevant to their condition [7, 11]. When accurate and representative information and guidance are provided, this can promote shared decision-making [12], and achieve more realistic and patient-specific expectations for functional and aesthetic outcomes following treatment.

Furthermore, people with HNC and their families express a desire and a need for tangible and appropriate, patient-centred information and resources to include the long-term lived experience following treatment [13]. HNC patients find it difficult to imagine life after treatment and value opportunities to learn how others coped when confronted with a similar situation [7]. Patient-centred information and patient experience narratives could greatly augment the information patients’ receives before and after treatment, potentially improving coping and adaptation, as well as promoting satisfaction with the delivery of care [14]. Patient experience narratives are recognised as central to UK health policy and have an important role in supporting shared decision-making and improving health [15].

With the increased availability of the internet, most patients, relatives and carers can assess health information via web-based resources to inform decision-making about healthcare options [16]. It is important to scope the availability and nature of web-based interventions for patients with HNC, to assess their quality and to map the evidence on how patients use these resources to aid preparedness for treatment. The aim of this scoping review was twofold: (1) map current websites available for HNC patients and assess their quality, and (2) identify published research papers and provide a summary of current evidence, to include gaps in knowledge [17] on utility of web-based interventions for patients with HNC. Such information would prove helpful to inform future online intervention development for this patient population.

Two research questions were developed as a focus for the scoping review:

(a)What web-based interventions are available for patients with HNC to aid decision-making and preparedness for treatment?

(b)From empirical studies, identify and review how HNC patients use web-based interventions across the treatment trajectory.

## Methods

Scoping review methodology is evolving and inherently beneficial for examining broad areas, being particularly useful for reporting on the types of evidence which may inform practice or identify key gaps in the evidence [18]. Reviews of this nature are also particularly useful for mapping a topic in order to communicate the breadth and depth of knowledge in the field, especially when study designs are expected to be heterogeneous [19]. Scoping reviews are also valuable when synthesis involves non-research material [20] such as websites. They differ from traditional systematic reviews in that they do not tend to inform formal quality assessment of empirical evidence [21]. A scoping review was deemed appropriate to meet the aims of this study and particularly fitting for this topic, therefore enabling authors to provide a description of the ‘extent, range and nature’ [21] of the available evidence on web-based resources for HNC patients, setting this in context in terms of current understanding, alongside identifying gaps to inform future research and web-based intervention development.

A protocol was developed to guide this scoping review but has not been published (Online Resource 1, supplementary information). This scoping review is compliant with the Preferred Reporting Items for Systematic Reviews and Meta-Analyses extension for scoping reviews (PRISMA-ScR) [22] recently published to support authors in preparing and reporting of scoping reviews [23]. These authors [23] advocate using this new guidance alongside the well-established and detailed Joanna Briggs Institute (JBI) Methodological Guidelines [24] for the conduction of the scoping review, both of which were used to guide and frame this review.

To ensure no reviews had previously been published in this field, an initial search using the search terms (Web-based interventions) AND (head and neck cancer or oral cancer or oropharyngeal cancer) AND (scoping review OR scoping studies OR systematic review OR literature review) across seven electronic databases (subsequently used for this scoping review) was undertaken. In addition, the Cochrane Database of Systematic Reviews and PORSPERO and JBI Systematic Review Register were searched using the search term (head and neck cancer), yielding no previous reviews related to web-based interventions for this patient population. This suggests that such a review is timely, in light of the increasing development of web-based interventions across healthcare settings, to include this patient population.

### Search strategy

For the purpose of this review, web-based interventions were operationally defined as ‘a primarily self-guided intervention programme that is executed by means of a prescriptive online programme operated through a website as sources of health information for patients’ [25]. As this scoping review melded empirical literature and websites, a separate search strategy was required for each type of evidence.

#### Empirical studies

An electronic search of the empirical studies was undertaken in Medline Ovid; Scopus; PubMed; Embase; Cochrane; Web of Science and PsycInfo, covering the years January 2010 to December 2020. The literature search was undertaken using the following keywords and subject heading terms: (see Table 1) S1: Web-based interventions + head & neck cancer, S2: Web based interventions + head & neck cancer + diagnosis & treatment and S3: Online resources + head & neck cancer. This review considered a variety of methodologies for the empirical studies, including qualitative and quantitative designs, alongside mixed methods studies. Guided by the JBI, inclusion and exclusion criteria were determined by population type, concept (outcomes) and context (intervention) framework (see Table 2).

**Table 1:** Search terms—empirical studies

Feb 2 2021	CINAHL	Medline Ovid	Scopus	Embase	WoS	Cochrane	PubMed	PsycInfo
S1 Web-based interventions AND head & neck cancer	07/392	Limiters: Oropharyngeal; laryngeal; mouth; head and neck neoplasms Cancer patients Quality of life Web-based interventions 3/16	5/15	0	6/22	1/4	2/26	1/17
S2 Web based interventions AND head & neck cancer AND diagnosis & treatment	0	Limiters: Pharyngeal; oropharyngeal; laryngeal; mouth; head and neck neoplasms Surgery, reconstructive Cancer patients Quality of life Web-based interventions 0/2	0/1	0	0/2	0/1	0/5	0/7
S3 Online resources AND head & neck cancer	0	Limiters: Pharyngeal; oropharyngeal; laryngeal; mouth; head and neck neoplasms Surgery, reconstructive Cancer patients Quality of life 0/41	5/32	1/3	1/29	0/1	6/36	0/3

**Table 2:** Inclusion/exclusion criteria for empirical studies

Inclusion criteria
*Population* Patients 18 years and over, with HNC* *Concept* Primary research studies (any design) investigating the use of web-based interventions designed for HNC patients aiding decision-making and preparedness for treatment, including the effects *Context* The web-based interventions must include some specific content for HNC patients. There were no limitations on the type of intervention or its duration, as it was not the purpose of this review to examine the interventions themselves* Other inclusion criterion: • Published in the English • Full-text peer-reviewed publication • Published between 2010 and 2020 to ensure the publications were relevant, given the rapid development of web-based information
Exclusion criteria
• Case reports, opinion pieces or letters • Studies were patients with HNC were solely palliative/end of life • Studies relating only to partners/caregivers of HNC patients

The search was carried out on 2 Feb 2021. The titles and abstracts of the research papers were initially screened (RK) to identify potentially eligible papers and any areas of uncertainty were resolved by another reviewer (CJS). The full manuscripts of potentially eligible papers were further independently screened against eligibility criteria by two reviewers (RK and CJS), with a third reviewer (PG) available to resolve any conflicts of opinion but not required. This determined a definitive list of included studies (Table 3). No additional hand searching was conducted but references of the included papers were also screened for any other relevant papers that might have been missed by the search.

**Table 3:** Study characteristics of empirical studies

Citation	Country of origin	Study population	Concept	Context	Design	Key finding related to research question
Badr et al. [12]	USA	Oral cancer survivors who had completed radiotherapy within the last 12 months and lived with a spouse/ partner or other family member who served as their primary caregiver	A web-based intervention to improve survivor self-management and survivor/caregiver QOL	Patients who have completed treatment for head and neck cancer and their caregivers	Grounded Theory methodology	Survivors wanted to learn from other survivors about what self-care strategies worked and did not work
Biggs et al. [37][36]	UK	Educational websites (patient.co.uk and wikipedia.org) and cancerresearchuk.org	Comparison of three cancer websites including head and neck cancer information	Specific websites and their resources, including those for head and neck cancer	Website assessment scoring; Factorial content across eight domains and ease of reading score	Patients should be encouraged to access health professional maintained sites
Brady et al. [38]	UK	Professionals from head and neck cancer service and 5–15 patients	A pre-treatment care pathway for patients with head and neck cancer	An evaluation of a specific head and neck cancer service with staff and patients	6-stage experienced-based co-design approach	Patient experience videos and a buddy system for radiotherapy patients were highlighted
Cnossen et al. [39]	Netherlands	Patients recruited from the Dutch Patient Society for Laryngectomees. Healthcare professionals recruited from a multidisciplinary expert team at the VUmc	Designing a self-care programme for laryngectomy patients	Laryngectomy patients	Participatory design approach	Providing eHealth can be cost-effective, improve quality of life and have beneficial effects on health literacy, decision-making, healthcare participation, psychological well-being and physical activity levels
Duman-Lubberding et al. [42]	Netherlands/UK	Healthcare professionals from a multidisciplinary team involved in the care of HNC patients in one centre	HCP’s views on OncoKompass to monitor QoL in cancer patients	HCPs working with head and neck cancer patients	3-stage design using interviews, prototype development and cognitive walk-throughs	Tailored feedback and personalised advice on supportive care options could be an alternative solution to meet cancer survivors’ individual needs
Fallon et al. [33]	USA	A free, internet-based social networking site available to all cancer survivors and caregivers, worldwide	An evaluation of the purpose and use of an online website run by the American Cancer Survivors Network	Users of the CSN website	2-phase evaluation process	Experience-based knowledge and social support from other cancer survivors and caregivers offer informational and emotional social support
Malik et al. [37]	UK	A search of YouTube™	Assessment of YouTube™ videos and evaluation of user responses	Videos relating to laryngectomy on one online platform	Quantitative analysis of metrics and thematic analysis of a purposive sample of videos	The use of online resources as part of supported decision-making prior to surgery
Manne et al. [35]	USA	Disease-free HNC patients from single US state	Development and pilot study of a web-based self-care management tool for oral cancer patients	Patients who are currently cancer-free and have access to a computer	Intervention content guided by social cognitive theory. Single-arm pilot study of intervention	The intervention may have a beneficial impact on self-efficacy, preparedness for survivorship care and QOL
Peterson et al. [43]	USA	HNC patients in one US centre	Evaluation of a home monitoring system to identify dehydration in patients receiving radiotherapy treatment for HNC	Patients receiving a specific treatment modality in one cancer centre	A feasibility study of the intervention including clinical review of metrics and a patient questionnaire	The use of health information technologies may alter how healthcare providers and patients interact with personal health information to enhance clinical decision-making
Saroa et al. [41]	Canada	Post-treatment HNC patients aged 18–65 years	To determine the information needs of HNC patients following treatment	Patients in one area of Canada who have completed treatment for HNC cancer	A self-administered survey	Participants identified information about survivorship as most important
Schwarzbach et al. [31]	USA	Search engines—Google, Yahoo, and Bing	Analysing online information about HNC	Functional websites written in the English language identified from 3 search engines	A cross-sectional website analysis with validated tools to measure quality and readability	Misleading or confusing websites can compromise medical decision-making and jeopardise the patient-physician relationship

#### Websites

The search terms used to identify the first 100 unique websites relating to HNC, accessible through one search engine (Google), are detailed in Table 4. This was to identify free, open-access web-based interventions available to patients with HNC. The review was not limited to any specific setting (community, hospital) and there were no geographic limitations.

**Table 4:** Search terms—websites

Search engine	Total found	Total screened	Excluded post screening	Reasons for exclusion	Added	Reason for addition	Final No
Google	1,730,000	105 unique sites	31	Sites not found Security issues flagged Duplication	1	Identified on another site. Not found during Google search	74
Google		74	12	For health professionals only			62
Google		62	30	Contained only advertisements, media reports/research findings, clinical guidelines, blogs			32

Previous work exploring how the public used the internet to search for health information indicated that people were only likely to look at the first ten unique sites [26]. However, subsequent authors reviewing websites have elected to expand the number of sites examined in the interests of rigour, with between 50 and 120 sites generally screened [27–29]. For the purposes of this review, the first 100 unique sites were identified on 3 February 2021 by the first author (RK), while logged into the Google search engine, saved for analysis (Table 5).

**Table 5:** Characteristics of included websites

Link	Content	Key finding related to research question	Analysis HoN	QUEST score (range 0 (lowest) to 28 highest) + %
National Health Service (UK) www.nhs.uk/conditions/head-and-neck-cancer	Information on symptoms, causes, diagnosis, treatment, living with and complications	Preparedness for treatment		13(46%)*
Macmillan Cancer support (UK) www.macmillan.org.uk/cancer-information	Information on symptoms, causes, diagnosis, treatment, living with and support	Preparedness for treatment	HoN certified	
Cancer Research UK www.cancerresearchuk.org	Information on symptoms, causes, diagnosis, treatment, living with and support	Preparedness for treatment		19 (68%)
National Cancer Institute (USA) www.cancer.gov/types/head-and-neck	Information on symptoms, causes, diagnosis, treatment, research and coping with cancer	Preparedness for treatment		15 (54%)
Wikipedia www.wikipedia.org/wiki/Head_and_neck_cancer	Information on symptoms, causes, diagnosis, prevention, management, prognosis, epidemiology, research, external links	Text only		23 (82%)*
Wikipedia (Wikidoc) www.wikidoc.org/index.php/Head_and_neck_cancer	Information on classification, history and symptoms, differential diagnosis, primary prevention. External links to Q&A, treatment and radiology info	Text only		19 (68%)
National Institute for Health and Care Excellence (UK) www.nice.org.uk	Guidance (27) advice (1) pathways (6) local practice (2) news (6) quality standards (1)	Preparedness for treatment		26 (93%)
Centers for Disease Control and Prevention (USA) www.cdc.gov/cancer/headneck	Information on symptoms, causes, prevention, diagnosis, treatment, clinical trials, rehabilitation, support	Preparedness for treatment		21 (75%)
American Society of Clinical Oncology www.cancer.net	Information and support	Questions to ask	HoN certified	
Oxford University Hospitals NHS Foundation Trust www.ouh.nhs.uk/cancer/cancer-by-type/head-and-neck	Details of service personnel and services at Oxford University Hospital. Links to other sites for information and support. Large selection of patient information leaflets	Preparedness for treatment		12 (43%)*
Head and Neck Cancer UK www.hancuk.org	Patient stories; information and support	Preparedness for treatment Patient narratives (text only)		4 (14%)
The Christie NHS Foundation Trust www.christie.nhs.uk	Information and support; services, PALS	Preparedness for treatment		14 (50%)
Radiological Society of North America www.radiologyinfo.org	Radiation information for patients. Sub section provides information on various options for radiation therapy in HNC	Preparedness for treatment	HoN certified	
The Royal Marsden NHS Foundation Trust www.royalmarsden.nhs.uk	Details of services and information and support. Dedicated head and neck page	Preparedness for treatment		8 (29%)
Head and Neck Cancer Alliance (USA) www.headandneck.org	Survivorship webinars/videos; information and support	Patient narrative videos( not specifically about preparedness)		18 (64%)
British Association of Head and Neck Oncologists www.bahno.org.uk	Professional organisation with patient area	Information on prevention and symptoms		14 (50%)
British Columbia Cancer (Canada) www.bccancer.bc.ca	Links to a number of guides for patients attending the centre. Dedicated page for HNC with information and support links	Preparedness for treatment	HoN certified	
Merck (Germany) www.merckgroup.com	Company webpage available to the public. Several patient experience videos and infographics on site	Preparedness for treatment Patient narrative videos (not related to preparedness)	HoN certified	
Cancercare (USA) www.cancercare.org/diagnosis/head-and-neck-cancer	Information on support and information including podcasts, workshops and publications. Board for questions to be submitted and general topic info. Links to other organisations and webpages	Preparedness for treatment		16 (57%)
Cancer Council NSW (New South Wales, Australia) www.cancercouncil.com.au/head-and-neck-cancer	Info on subtypes (very good illustrations). Links to other support and information	Preparedness for treatment	HoN certified	
Moffitt Cancer Center (USA) www.moffitt.org/cancers/head-and-neck-cancer/faqs	Information on diagnosis, treatment, FAQs. Patient experience videos	Preparedness for treatment Patient narrative videos (not related to preparedness)		10 (36%)
Cancer Council Victoria (Australia) www.cancervic.org.au	Links to information on overview, diagnosis, treatment	Preparedness for treatment	HoN certified	
Emory University (USA) www.cancerquest.org/patients/cancer-type	Link to HNC webpage for patients. Developed in consultation with patients. Information on anatomy, types, risk factors, symptoms, diagnosis, staging, treatment, survivorship, international resources (links to services across the world)	Preparedness for treatment		16 (57%)
Memorial Sloan Cancer Center (USA) www.mskcc.org/cancer-care/types/head-neck	Information on signs and symptoms, diagnosis, treatment, survivorship	Preparedness for treatment		13 (46%)
American Association for Cancer Research www.aacr.org/patients-caregivers/cancer/head-and-neck	Information on subtypes, survivorship videos and patient advocacy section	Preparedness for treatment Patient narratives (text only related to survivorship)		10 (36%)*
Chris O’Brien Lifehouse (Australia) www.mylifehouse.org.au/for-patients/cancer-types	Covers different cancers. HNC page covers diagnosis, treatment, support. Tab for patients/caregivers. Includes links to support groups and beyond five page	Preparedness for treatment		14 (50%)
Cancer Council (Australia) www.cancer.org.au	Sections on Talk to Someone (support groups, telephone peer support), Cultural Resources, Practical Assistance, Emotional Assistance, Online support services including e-learning	Preparedness for treatment	HoN certified	
Irish Cancer Society www.cancer.ie/mouth-head-and-neck-cancer	Information on diagnosis, treatment, support and publications	Preparedness for treatment		16 (57%)
Fox Chase Cancer Center (USA) www.foxchase.org	Patient and families support services section. Page on HNC including diagnosis, treatment, survivorship, patient stories and clinical trials	Preparedness for treatment Patient narratives (text only, no HNC patients featured)		16 (57%)
American Head & Neck Society www.ahns.info/resources/education/patient_education	Patient education from the American Head and Neck Society includes survivorship, interviews, HPV vaccine for boys, find a physician	Patient narrative videos with cancer survivors reflecting on cancer journey		15 (54%)
ENT Health (American Academy of Otolaryngology—Head and Neck Surgery) www.enthealth.org/conditions	HNC page covers symptoms, treatment, questions for doctors	Preparedness for treatment		13 (46%)
Head and Neck Cancer Australia www.headandneckcancer.org.au	Website dedicated to HNC for patients/families/professionals. Tabs cover: cancer types, diagnosis, treatment, health and well-being, caregivers/family/friends, find support, webinar, personal stories, find a clinic, latest news. Additionally for health professionals includes patient information sheets and research info	Preparedness for treatment Patient narratives (text only)		13 (56%)

^*^Agreed QUEST score by RK and CJS

### Data extraction and analysis

#### Empirical studies

Data was extracted using a specially designed data extraction tool (Online Resource 2, see supplementary information) on an Excel spreadsheet by one member of the research team (RK) and checked by a second reviewer (CJS) to achieve consensus. Data was mapped out in a descriptive manner according to the following: author, year of publication, journal, database, search terms, design, outcomes and final inclusion. Following the data extraction, the studies were categorised based on their findings, and then themes developed and revised accordingly, following review and discussion by members of the team (RK and CJS). This process was guided by Thomas and Harden’s 3-step thematic analysis framework, with four final agreed themes: (1) information about HNC and related treatment, (2) advice and support during HNC treatment, (3) management strategies and adjustment to life with or beyond HNC and (4) optimisation and quality of web-based interventions for HNC patients.

Due to the wide range of different study methodologies, and in keeping with the accepted remit of the scoping review [24], specific quality appraisal was not conducted on each study. Instead, key study limitations, having been documented, were extracted to inform the synthesis of data within themes.

#### Websites

Following screening of the websites against the inclusion and exclusion criteria (*Table 2), data was extracted from 32 eligible websites which were patient focused and included specific areas for HNC patients (Online Resource 2, supplementary information). Websites were checked using the Health on the Net [30] toolbar on a web browser to identify those which had been certified by HoN [31]. HoN promotes useful and helpful health information online, providing voluntary certification for presentation of reliable, transparent information [30]. Given that only eight of the 32 websites were HoN certified a decision was reached to critically appraise [22] all of the websites using QUEST (Online Resource 3, supplementary information), a validated tool for review of websites [32]. The websites were scored by RK using QUEST (*n* = 32) with 20% of the sample independently analysed by CJS (*n* = 6) to ensure concurrence. Joint QUEST scores ranged from 10 to 23 (Table 5). A separate readability assessment was not undertaken.

## Results

The final set of relevant empirical studies is presented in tabular form using the data presentation tool (Table 3). This tool includes commentary on how the results relate to the research questions. The final list of included websites (*n* = 32) scored using QUEST/HoN is presented in Table 5.  Findings of both sets of data is aligned to the study aim and questions of this review.

### Empirical studies

#### Range of studies

**Fig. 1:**
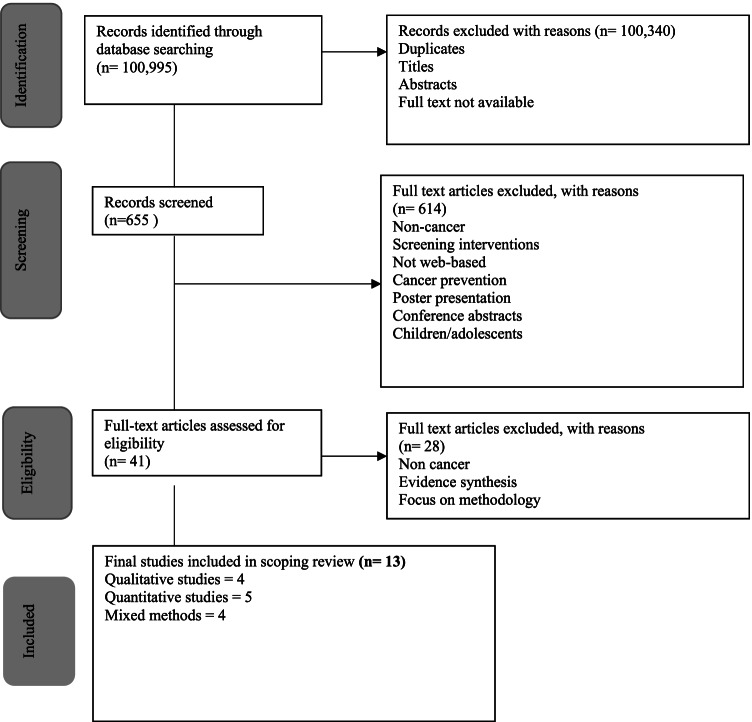
PRISMA-ScR

Eligibility screening of 655 papers resulted in 38 papers meeting the inclusion criteria for review of full text (Online Resource 2, supplementary information). Following review of full text by RK and CJS, 13 papers were selected for the final scoping review as detailed in the PRISMA-ScR reporting tool (Fig. 1). Papers originated in the USA (*n* = 6) [12, 31, 33–35], UK (*n* = 3) [36–38], Europe (*n* = 2) [39, 40], Canada (*n* = 1) [41] and a collaborative study (*n* = 1) [42]. Most of the included studies focused on the process of intervention development and testing (*n* = 8) [12, 34, 35, 38–40, 42, 43], whereas five studies determined the quality of patient information within the web-based interventions (*n* = 5) [31, 33, 36, 37, 41].

The majority of the web-based interventions focused solely on the post-treatment component of the treatment trajectory, ranging from exercise programmes [34], rehabilitation programmes [39] to self-management and survivorship initiatives [12, 35, 40, 42]. One was remote monitoring of symptoms, namely dehydration risk during treatment [43], and one focused solely on the pre-treatment period [38]. Some studies that focused on online intervention development assessed e-literacy and readability [31, 36], alongside information needs and preferences [41], while others explored HNC patients’ use of online resources such as YouTube™ [37] and online cancer networks [33].

Studies focusing on web-based development [12, 35, 42] or adaption of current interventions [34] used a range of research designs including qualitative [12], quantitative methods [36, 37] to include surveys [33, 41], randomised controlled trial [40]; and mixed methods for a feasibility study [43].

Participant sample size within studies focusing on intervention development or evaluation ranged from 14 [38] to 4295 [33]. Studies providing a review of HNC web-based resources, ranged in number of those being reviewed from 3 [36] to 27 [31] to 96 online videos [37]. Within this scoping review, six studies focused purely on patients [33–35, 40, 41, 43]; one focused on both patients and family carers [12] and three on patients and healthcare professionals [38, 39, 42]. The remaining three papers [31, 36, 37] explored websites which could be used by patients, carers or healthcare professionals.

#### Themes

### Theme 1: Providing information about HNC and related treatments

Overall, very few studies provided information on the pre-treatment phase of the HNC trajectory, with one of the papers addressing the preparedness of HNC patients for surgery, which was specific to those undergoing a laryngectomy [37]. Findings for this targeted population revealed the potential benefit of patient videos as a mechanism for providing information to aid decision-making surrounding having a laryngectomy, and gaining insight into the perceived post-treatment QOL issues. While this study demonstrated a demand for patient-focused information on treatment of laryngeal cancer, a sizeable patient population was attempting to meet this need through YouTube; with the most beneficial, reliable and detailed videos being those produced by healthcare professionals [37].

Online resources developed for healthcare professionals had more detailed and accurate information that those uniquely for a patient cohort [36, 37]. Subsequently, open-access professional web-based interventions may expose patients to alarming information or excessively complex information, with the possible result of evoking distress and adversely influencing patients’ behaviour and decision-making.

When both quality and readability of educational content from HNC web-based interventions have been examined, the consensus drawn is patients should be advised to access online sites maintained by healthcare professionals to ensure accuracy of information [36]. Nonetheless, this premise cannot be universally accepted, given evidence that online information about HPV +ve oropharyngeal cancer was found to be insufficient following quality assessment of 27 unique web pages [31], using the QUEST analysis tool [32]. The readability of these 27 websites was also poor, reflecting the challenges of writing information about complex conditions in a way that is easy for the public to understand. Misleading or confusing information on web-based interventions can compromise decision-making and jeopardise the patient-physician relationship. Creating a few trustworthy web-based resources, that meet quality criteria and are routinely reviewed, could result in the provision of a list of these vetted websites being given to patients and published on a hospital/clinic’s own web page.

### Theme 2: Ongoing advice and support during HNC treatment

This was the predominant focus for many of the studies (*n* = 7) [12, 33–35, 39, 40, 43]. Healthcare professionals have suggested that self-care interventions for HNC patients should include problem-solving advice to help patients when they return home [12, 34, 40], whereas patients would argue that potential problems should be identified before treatment, so they have time to think about actions they may need to take, and questions that they may want answer [39]. Although online self-care programmes can be helpful for HNC patients [39], adherence may depend on the degree of connectivity patients feel with the team who are treating them [35] and support offered by healthcare professionals, in combination with an online resource [34]. Discussing and promoting specific online resources with patients during consultations may add value to their use as a supportive interventions for patients at home [42].

Some web-based interventions were developed to track and monitor patient’s symptoms at home, by collecting biometric parameters remotely, potentially facilitating closer partnership with clinicians for monitoring care, promoting engagement and shared decision-making [43]. Monitoring physical and physiological parameters remotely and in real time, including pulse, blood pressure and weight, plus patient-reported outcomes may offer cost-effective strategies to optimise cancer care outside of the clinic setting. Such interventions may also support patients by offering reassurance that their healthcare professionals are being provided with objective data during active treatment and alerted to any potential health-related issues, such as the aforementioned, which demonstrated early detection of dehydration during radiotherapy [43]. Ease of access to remote monitoring may also encourage patient adherence to treatment, with reporting of new symptoms or side effects more readily, thus improving real-time clinical decision-making.

In most of the studies where web-based self-management interventions were tested, patients valued the knowledge gained, enhanced control and perceived self-efficacy from using the intervention [35, 39, 43].

### Theme 3: Management strategies promoting adjustment to life with or beyond HNC

Within web-based interventions, use of former HNC patients to describe their long-term lived experience is a method used by some researchers to provide a realistic and patient-centred perspective. This provides an opportunity to model behaviours and share survivor stories of beneficial self-management interventions [12, 34, 38]. Furthermore, some researchers found that including patient experience stories within an online intervention provided other survivors with a sense of emotional and social support [33]. This was particularly true for HNC patients who had less social interaction because of increased self-consciousness related to their altered appearance [33]. Even those who are regarded as longer-term survivors indicated an interest in learning self-care strategies from others [12]. Overall there was a clear acknowledgement, derived from patient feedback, that more patient stories/videos were advocated to enhance usefulness of web-based interventions for HNC patients [12, 38]. Nonetheless, HNC survivors recognised the need for caution when accessing patient narratives, reflecting the uniqueness of an individual’s journey.

### Theme 4: Optimisation and quality of web-based interventions for HNC patients

From the 13 included studies, the barriers and enablers identified when designing and using web-based interventions for HNC patients are noted in Table 6. To promote acceptability and usability of web-based interventions, most authors advocate the need for extensive stakeholder engagement, in an iterative process, to minimally include HNC patients and clinicians [39, 43]. Involving patients and clinicians throughout all aspects of web-based intervention development and the testing process can enhance the acceptability and effectiveness of web-based interventions [39]. Furthermore, consideration should be given to the quality (reliability and accuracy) and readability of the information when designing online material [31].

**Table 6:** Enablers and barriers to designing and using web-based interventions for HNC patients

Enablers	Barriers
Theoretical framework	Poor quality information
Co-production	Poor design features
Stakeholder engagement	Lack of signposting—poor navigational tools
Ease of access	Poor health literacy of patient
Experience-based information	
Health literacy considered	
Credibility of developers	
Based on current evidence	
Tailoring/layering of information	

Some studies evidenced patients’ desire for web-based interventions to be interactive so that patients/carers could actively engage with healthcare professionals in real time or submit questions [12]. This would require sites to be monitored by staff, which is likely to be appropriate if the premise of the intervention is for symptom tracking and monitoring, as a mechanism for directing care delivery. However, for other aspects of survivorship care, self-management applications where patients can use them independent of healthcare professionals may facilitate sustainability of long-term survivorship care [40]. Furthermore, if HNC web-based interventions can be tailored to reflect individuals’ needs, this is likely to enhance health-related QOL [37, 38, 40, 42].

### Websites

#### Range of sites

Following eligibility screening using the inclusion/exclusion criteria, 32 websites were included in the final analysis. The websites were generated in a range of countries, namely, US (*n* = 13), UK (*n* = 9), Australia (*n* = 5), Europe (*n* = 2), mixed (*n* = 2) and Canada (*n* = 1). Sites tended to be owned by charities (indicated by request for donations) (*n* = 16), followed by government (*n* = 8), healthcare facilities (*n* = 5), commercial organisations (*n* = 1) and free encyclopaedia (*n* = 2). The content of the website has been themed to include patient stories, information on the management of HNC and information on healthcare professionals.

#### Themes

### Theme 1: Patient stories

There was a combination of tumour-specific (HNC) and general cancer websites identified, which encompassed patient stories at key points of the cancer continuum, such as at diagnosis, during treatment and living with and beyond cancer [www.macmillan.org.uk; www.ahns.info; www.headandneckcancer.org.au]. Some websites had navigational tiles, clearly indicating the location of patient stories within the website, which generally made access much easier [www.hancuk.org; www.headandneck.org]. Others provided enhanced navigation by adding a short summary descriptor of video content (tumour subtype and aspect of cancer journey) [www.merckgroup.com], making it easy for viewers to select videos likely to best meet their needs. Within some of the generalist cancer websites, indexing of tumour type was not provided [www.cancercare.org], which could lead to difficulties for HNC patients to find the most relevant and meaningful stories for them.

Websites varied on mode for displaying patient stories, from text form [www.hancuk.org; www.christie.nhs.uk] to videos [www.cancercare.org; www.headandneck.org; www.merckgroup.com] or webinars [www.headandneck.org]. Some websites include blogs [www.macmillan.org.uk], enabling people to enter journal excerpts about their experiences, and podcasts [www.cancerresearchuk] for patients to speak about specific aspects of their cancer journey. As before, when there was no signposting to blogs and podcasts for specific cancer types, viewers were required to trawl through all the available content, making navigation potentially cumbersome.

Most of the Australian sites featured in this review [www.cancercouncil.com.au; www.cancervic.org.au; www.cancer.org.au] used the Australian Cancer Council branding which lends a certain familiarity, authenticity and credibility for viewers around cancer information. One website had a facility for interactive patient engagement [www.cancer.org.au], with a tab entitled *iheard* which explores the myths around cancer and allows viewers to submit questions about things they may have heard or been told. Other innovative additions to the patient stories within websites were provision of a side bar for each story which included disease-specific information, the doctors involved in the patient’s care and what type of treatment the patient had [www.foxchase.org]. This was provided by an American healthcare organisation website, thus inclusion of the doctors’ details facilitated individuals’ visibility, which could potentially promote onward patient referral, within a private healthcare setting. Furthermore, one site included a *Survivor Support* tab which can put newly diagnosed patients in touch with cancer survivors to provide one-to-one support [www.cancer.ie].

### Theme 2: Information on the management of HNC

Written patient information is available on most of the sites with the exception of www.enthealth.org. Some websites include information on prevention of HNC [www.cdc.gov], treatment options [www.macmillan.org.uk; www.ahns.info; www.headandneckcancer.org.au], emotional support [www.cancer.gov; www.cancer.net; www.bccancer.bc.ca; www.mylifehouse.org.au], symptom management such as pain [www.bccancer.bc.ca], and spiritual care [www.bccancer.bc.ca], prognosis [www.wikipedia.org; www.cancerresearchuk] and links to other patient information resources rather than providing them directly [www.cdc.gov; www.wikipedia.org; www.wkidoc.org]. Many of the sites include information about current research [www.macmillan.org.uk; www.cancerresearchuk; www.cancer.gov], which could be regarded as an incentive for viewers to make a donation to the site.

Of note, there were websites identified that focused on clinical guidance and treatment pathways designed for healthcare professionals [www.nice.org.uk], but can be accessed by the public. Such sites may help patients to see what is considered best practice, but patients may not be aware that not all guidance will be adopted in every health authority, which has the potential to cause confusion and anxiety. Other websites were clearly aimed for professionals but one such site also included a tab entitled *Patient Area* which provided a series of external links for further advice and support [www.bahno.org.uk]. One of the links included was to a cookbook specifically for HNC patients, which describes the challenges and tip for eating and swallowing after surgery and a series of recipes from HNC survivors.

Some websites pertained to specific healthcare organisations [www.ouh.nhs.uk; www.christie.nhs.uk; www.royalmarsden], which more often listed HNC as one of many cancer types. These websites aligned to healthcare organisations varied immensely on the amount and quality of patient information on HNC, directly reflected by QUEST scorings (see supplementary information). On addition to the information in text form, one of the websites [www.royalmarsden] provides a tab titled *Patient Information Library*, with a recite button enabling an audio guide, which increases accessibility for those with impaired vision or illiterate. In contrast, some of the titles of the navigational tiles are quite complex, requiring patients to know the clinical name of tests, treatments, etc. in order to access the correct information.

### Theme 3: Information about the healthcare professionals/HNC team

Information about the healthcare teams often appears on the organisational websites, with some being simply a list of the team members [www.ouh.nhs.uk; www.moffitt.org], while others include staff pictures and key contact details [www.christie.nhs.uk; www.mylifehouse.org.au]. Websites include videos from members of the HNC team, often relate to professionals advising on treatment modalities specific to their professional discipline, such as a radiographer detailing radiotherapy [www.ouh.nhs.uk], surgeons talking about what to expect following an operation [www.mylifehouse.org.au] and a radiologist outlining diagnostic tests [www.radiologyinfo.org]. This latter site provides specific information using text, videos and images which could be considered a particularly helpful way of illustrating to patients what some of the diagnostic equipment looks like.

## Discussion

One consequence of the COVID-19 pandemic has been the increasing reliance on electronic communication, with greater patient acceptance surrounding the need for virtual communication strategies [44]. As patients increasingly look to the internet for advice and support, healthcare professionals are in a position to provide high-quality, evidence-based online interventions. These web-based interventions have the potential to aid decision-making, provide advice, promote self-management and enable real-time remote monitoring of symptoms [45, 46]. With this in mind, the main findings of this scoping review was the lack of evidence-based and theory-driven web-based interventions, encompassing HNC patient’s lived experience for use in clinical practice to aid decision-making and preparedness for treatment. More often web-based interventions were not co-produced by professionals and patients.

### Aiding shared decision-making

There is an evident gap in both the literature and availability of high-quality patient-focused online resources for use in clinical practice to aid decision-making for HNC patients. Studies within the scoping review acknowledged that patient narratives would be particularly helpful in web-based interventions for this complex tumour group but are often absent [12, 38]. When available and accessible on online platforms such as YouTube™, this information proved helpful but limited to a subsection of HNC patients (those with a laryngectomy) [37], therefore not addressing the wider needs of this patient population. Given the specific, often long-term impact of various HNC treatment options [38], this necessitates the need for web-based resources that provides information across the spectrum of treatment options with patient narratives embedded, which could be used to foster a shared decision-making approach [16, 47]. Multimedia approaches should be integrated for the sharing of patient lived experience, as studies demonstrate that patients do respond positively to such approaches [48], and could consequently augment face to face consultations by providing realistic patient-focused perspectives.

Cognisance should be given when developing web-based interventions on open-access platforms, as by definition they allow patients to find and engage with material freely, especially if the resource is not primarily intended for patients but healthcare professionals. Digital interventions for healthcare professionals entail predominately explicit facts and opinions, correlated with healthcare delivery systems and biomedical research, which can have a prescriptive focus [49]. Whereas patient-focused online resources, developed with embedded patient narratives, offer other patients substantial expertise on strategies for coping with issues relating to everyday living. These are often gained through trial and error of the lived experience, which can have added value as it is more easily comprehended and accessible for patients.

### Preparedness for treatment

Promoting physical and psychological preparedness for treatment is fundamentally important, which underpins many prehabilitation programmes [50], utilising behavioural change. Although integral to successful post-treatment outcomes, often patients find lifesyle behavioural modifications challenging [51]. Using web-based interventions can be helpful in motivating patients to make behavioural changes necessary to optimise treatment outcome [52, 53] but are unlikely to succeed if they are not accompanied by support or endorsement from their healthcare professionals [49, 54].

### Role in self-management and promoting positive coping skills

Self-management tools employed in HNC services [12, 35, 39] and more broadly in chronic illnesses [55] provide patients with coping mechanisms to facilitate autonomy, through voluntary actions. These can promote self-efficacy through building their personal skill base and facilitate relatedness through access to peer and other support services [12]. Other authors found that self-management tools improved self-knowledge and skills for HNC patients with tumour-specific concerns, but identified an issue surrounding the optimal timing of such interventions [40]. In the wider literature relating to chronic illness, partnership between patient-clinical teams was also identified as a key facilitator in self-management intervention success, but was also often the greatest barrier to success by its absence [56]. Therefore, to successfully promote the utility of online self-management programmes, these should be promoted by the clinical teams and discussed during subsequent follow-up appointments.

### Developing online interventions

Designing online interventions for patients presents a number of barriers and enablers relating to efficacy, credibility and quality (Table 6). Increasingly, it is recognised that developing online interventions should have a theoretical underpinning, for example, Theory of Self-Regulation [34] or Social Cognitive Theory [35], and intervention development guided by frameworks such as the Person-Based Approach [57] or experience-based co-design [38]. Despite having a theoretical basis, some online interventions were reported as less effective than hoped [40]. This does not mean there is no evidence of efficacy, but developers should undertake feasibility testing or process evaluation to ascertain what works, for whom and in what context. Such findings are integral, providing an opportunity for web-based intervention refinement before formal evaluation and widespread implementation.

Patient and healthcare professional engagement in intervention development should be regarded as essential and a key enabler toward making an online resource maximally effective, credible and ensuring that it has clinical utility. Employing intervention development frameworks such as the Person-Based Approach [56] or experience-based co-design [38] ensures key stakeholders and end-users are active participants at all stages of the online intervention development. While there was evidence of stakeholder engagement throughout all steps of intervention planning and optimisation in some studies within this scoping review [12, 35, 39], others limited participant engagement to testing the interventions [34, 40, 43]. This limited engagement was also evident in the broader cancer literature [16, 52, 58]. Although online interventions for HNC patients should be co-designed using multiple stakeholders, this has inherent challenges surrounding project management, budgeting and time investment. This is not unique to this patient population, nor to cancer care, but evident in the broader health literature [59, 60]. It would also appear that engaging patients and healthcare professionals in intervention development may depend more on the epistemological and ontological beliefs of the researchers, despite growing recognition of the importance of engaging the public in research [61, 62].

### Essential need to consider health literacy for the HNC population to promote engagement

It is well documented that there is a preponderance of HNC patients that preside from lower socioeconomic classes, with poorer health literacy. Health literacy is predicated on patients’ ability and motivation to access, understand and use information to promote and maintain health [63]. Further barriers to health literacy can be evidenced with poor quality information or poorly presented content, adding to patients’ stress and anxiety [63]. Furthermore, low health literacy is negatively correlated with the ability to discriminate between high- and low-quality eHealth information [64]. One of the criticisms of health-related websites is the poor quality of the information provided [65]. Compounding this issue, is the growing number of cancer patients who use online information to guide healthcare decisions, either for themselves or on behalf of another person. This has consequently led to the development of validated tools to evaluate and appraise online health information. Through the development of quality assessment tools such as HoN and QUEST, and tools for calculating ease of reading [31, 36], online health information can be assessed objectively for quality. We would recommend that patients should be directed to websites which have proven efficacious based on quality assessment tools, mitigating exposure to poorer quality and misinformation from other web-based resources [36]. Directing patients towards appropriate sites during consultations or navigating sites with them (which may result in increased time pressures) can provide additional reassurance and may improve the patient-professional relationship [66].

### Meaning of the study with possible implications for practice, policy and research

The development of high-quality, evidence-based and theory-driven online resources has implications for practice, aiding shared decision-making, preparedness for surgery, remote monitoring of symptoms and self-management. Policy makers should focus on improving guidance for development and quality assessment of such sites. Additionally, the quality assessment of public-facing websites providing health information needs to be more readily available. Finally, there are several implications for future research. Research on web-based interventions for patients should demonstrate co-production with patients and other key stakeholders to improve acceptability and usability. Web-based intervention development should have a theoretical underpinning to enhance the quality and effectiveness of such resources in clinical practice.

### Limitations

This scoping review focused specifically on web-based interventions for patients, thus excluding studies solely described for caregivers, which could also prove helpful to patients. Limiting studies to English text and focusing on those published between 2010 and 2020 may have excluded some valuable papers. However, setting such parameters enabled the capture of the most current studies in a rapidly evolving era of web-based intervention development.

In trying to mimic patient behaviour, we chose to review the first 100 unique websites which appeared when ‘head and neck cancer’ was entered into one search engine. As a result, some valuable websites may have been missed. Finally, this scoping review sought to understand the nature of online resources currently available and how patients interacted with them; therefore, a readability assessment was not performed, as it was not crucial component to this type of review.

## Conclusion

There is no evidence of other scoping reviews melding both research papers and websites to understand the breadth of web-based interventions currently available for HNC patients. Despite the increasing availability of online resources, there is a lack of high-quality, web-based interventions for HNC integrating patient narratives to aid decision-making and preparedness for treatment. Recognising the need for further work, this scoping review will provide a foundation to inform the planning and development of a web-based intervention, integrating patient narratives to promote patient preparedness for oral cancer treatment. Credibility and effectiveness can be promoted through use of co-production methodologies, underpinned by theoretical frameworks, which can enhance clinical utility of web-based interventions.

## Supplementary Information

Below is the link to the electronic supplementary material.

Supplementary file1 (PDF 392 kb)

## Data Availability

All data generated or analysed during this study are included in this published article [and its supplementary information files].

## Code availability

Not applicable.

Human participants were not recruited by any of the authors for this scoping review.
