# Supplementary material for: Availability and use of web-based interventions for patients with head and neck cancer: a scoping review
Source: J Cancer Surviv. 2022 Jan 28;17(5):1309–26. doi: 10.1007/s11764-022-01168-1 (PMC8794623; doi:10.1007/s11764-022-01168-1)
Supplement: Supplementary file 1 — Supplementary file1 (PDF 392 kb) [file 11764_2022_1168_MOESM1_ESM.pdf]

Online Resource 1

Article title: Availability and use of web-based interventions for patients with head and neck cancer: A Scoping Review

Journal: Journal of Cancer Survivorship

Authors: Kelly R, Semple CJS, Gordon P, & Thompson R .

Corresponding author: Semple CJS, cherith.semple@setrust.hscni.net

Scoping Review protocol

TITLE: Identifying the range, nature and use of web-based interventions for patientsundergoing treatment for head and neck cancer: A Scoping Review Protocol

Abstract

Objective: To identify the range, nature and use of web-based interventions for patients undergoing treatment for head and neck cancer

Introduction: People with head and neck cancer and their families want to be able to accesstangible and appropriate, patient-centred resources including the long-term lived experience following treatment. They value opportunities to learn how others coped when confronted with a similar situation. The paucity of evidence relating to the review of such resources suggests a scoping review of this issue is timely, in light of the increasing development of web-based resources and interventions in this patient population.

Inclusion criteria: (a)Primary research papers, describing web-based interventions for head & neck cancer patients > 18 years of age; English language; Full text; 2010-2020 publicationor (b) free, open access web-based interventions for patients with head and neck cancer.

Methods: Peer reviewed papers from several databases; First 100 unique websites on Google

Keywords: head and neck cancer; web-based intervention; online resources

Introduction

Globally, head and neck cancer (HNC) affects approximately 550,000 people annually (Fitzmauriceet al 2017). The incidence is increasing in the UK and aetiology is changing, for example, HNC is affecting younger people from more affluent backgrounds, due to increasing HPV-related disease. Oral and oropharyngeal cancer incidence rates in the UK have risen by a third in the last three decades (CRUK 2019). Treatments and outcomes vary according to tumour histology, site and stage. Complex treatment pathways can produce significant side-effects and consequently

impact individual's quality of life (QOL) (Semple & Killough 2014). Head and neck cancers (HNC) are classified into the following subsites: oral cavity, oropharynx, hypopharynx, larynx, sinonasal tract, and nasopharynx (Hakim et al., 2011). The choice of treatmentis dependent on the location and stage of the tumour. Current treatment modalities with curative intentare surgery, chemotherapy and radiotherapy or a combination approach (Hinni et al., 2012). The impact of surgery on oral function varies hugely depending on the subsite and proportion of the oralcavity being resected, and consequently effects social, work and personal relationships, which influences post-treatment quality of life (QOL) (Semple et al 2019).

Clinical teams can face ethical, emotional and practical challenges when endeavouring to convey realistic and relevant views on the side-effects and outcomes of the different surgical procedures available for the management of cancer to the head and neck area. Provision of this information is paramount, to promote shared decision-making.

In an era were cancer survival rates are improving, therefore more people living with the effects of HNC and its treatment, to include physical and psychosocial disruption and a diminished sense of self, it is increasingly important that patients receive person-centred care, support and stratified information relevant to their condition (Parahoo et al 2019, Semple et al 2019). The wellbeing of people with HNC and their families is often interdependent and carers also report the need for accurateand representative information and guidance.

People with HNC and their families express a desire and a need for tangible and appropriate, patient-centred resources to include the long-term lived experience following treatment. They find it difficultto imagine life after treatment and value opportunities to learn how others coped when confronted with a similar situation (Semple et al 2019). Patient-centred information and patient experiences narratives have a key role in facilitating coping and adaptation, promoting satisfaction with the delivery of care. Patient experience narratives are recognised as central to UK health policy and have an important role in supporting decision making and improving health (Seale 2016). Despite the increased accessibility of the internet to most patients, there are few evidence-based web-based interventions available with patient-centred information and patient experience narratives for individuals with HNC. If available, this could help aid decision-making and preparedness for potentially life-altering surgery. Furthermore, the quality of existing websites available to patients and their families with oral cancer are rarely subjected to quality assurance, are clinician-focused rather than patient-focussed, provide limited information on the long-term consequences and have little information on post-treatment QOL. The available online resources have also a relatively high average reading age making them inaccessible to many of those with HNC, due to a high preponderance of people with poor literacy levels from lower socio-economic backgrounds.

Review question/s

(i) What web-based interventions are available for patients with head and neck cancer to aidpreparedness for, and the consequences of treatment?

(ii) How do patients use web-based interventions to aid preparedness for, and consequences ofhead and neck cancer treatment?

Inclusion Criteria

Population

This scoping review will consider (a) all research studies that include patients 18 years and over, undergoing treatment for head & neck cancer, that describe web-based interventions for this population. For the purpose of this

review, the operation definition of interventions is:

a primarily self-guided intervention program that is executed by means of a prescriptive online program operated through a website (Barak et al. 2009) or evaluations of websites as sources of health information for patients

The studies must be in the English language, full text and published between 2000-2020. (b) The first 100 unique websites related to head and neck cancer, accessible through one search engine.

Concept

This review will include primary research studies and web-sites that describe web-based interventions designed for HNC patients. The web-based interventions must include some specific content for HNC patients. There are no limitations on the type of intervention or its duration as it is not the purpose of this review to examine the interventions themselves.

Context

The review will consider (a) primary research studies related to web-based interventions for HNC patients (b) free, open access web-based interventions available to patients with HNC. The review will not be limited to any specific setting and there will be no geographic limitations.

Types of sources

The review will consider qualitative, quantitative and mixed methods studies. Qualitative studies mayinclude, but not be limited to phenomenology, grounded theory, ethnography, qualitative description, action research. Quantitative studies may include, but not be limited to experimental and intervention study designs, such as randomized and non-randomized controlled trials). Mixed methods studies may include, but not be limited to surveys, metric measurement and descriptive analysis.

Methods

Initial search strategy to elicit if any previous reviews have been conducted in this field of interest An initial search using the search terms *Web-based interventions AND (head and neck cancer or oralcancer or oropharyngeal cancer) AND (scoping review OR scoping studies OR systematic reviewOR literature review)* across the same databases planned for the full scoping review revealed no suchstudies. In addition the Cochrane Database of Systematic Reviews and PORSPERO were searchedusing the search term *head and neck cancer* and the Joanna Briggs Institute Systematic ReviewRegister was searched using the term *cancer* when the term *head and neck cancer* elicited no reviews.No reviews related to web-based interventions for this patient population were found. This suggeststhat such a review is timely, in light of the increasing development of web-based interventions in thispatient population.

Principal search strategy

A search of CINAHL; Medline Ovid; Scopus; Pubmed; Embase; Cochrane; Web of Science;PsychInfo; Google will be undertaken (Appendix I).

Search terms: <u>*Papers*</u>

S1: Web-based interventions + head & neck cancer

S2: Web based interventions + head & neck cancer + diagnosis & treatmentS3: Online resources + head & neck cancer

Websites

S4: Head and neck cancer

Information sources

Study selection

Having demonstrated the current lack of rigorously developed and accessible web-based interventions containing narratives of peoples' lives before and after treatment for HNC, the inclusion/ exclusion criteria were developed to focus on web-based resources for this specific group of patients. The guidance issued by the Joanna Briggs Institute for conducting and reporting a Scoping Review will be followed and the updated guidance from the PRISMA group (Tricco et al 2018) will be used to illustrate screening and selection of the peer-reviewed papers.

Data extraction

Data will be extracted using the extraction tool illustrated in Appendix II. of the Protocol. This data will be reviewed by both authors to achieve consensus. The extracted data will include specific detailson the author, year of publication, the design of the study, the reported outcomes and the decision oninclusion/exclusion in the final review as agreed by both reviewers.

Data presentation

The final set of relevant papers will be presented in tabular form using the data presentation tool illustrated in Appendix III of the Protocol. This tool will include commentary on how the results relateto the research questions. A narrative summary will accompany the tabulated results aligned to the objective/questions of this review.

References

Badr H, Lipnick D, Diefenbach MA, Posner M, Kotz T, Miles B, Genden E. Development and usability testing of a web-based self-management intervention for oral cancer survivors and theirfamily caregivers. European Journal of Cancer Care 2016; 25: 806–821.

Biggs TC, Jayakody N, Best K, King EV. Quality of online otolaryngology health information.Journal of Laryngology & Otology 2018; 132: 560–563.

Brady GC, Goodrich J, Roe, JWG. Using experience-based co-design to improve the pre-treatmentcare pathway for people diagnosed with head and neck cancer. Supportive Care in Cancer 2020; 28:739–745.

Cancer Research UK (CRUK) [Internet]. 2019[cited 26 Mar 2019]. Available from: https://www.cancerresearchuk.org/health-professional/cancer-statistics/statistics-by-cancer- type/head-and-neck-cancers/incidence

Chang KL, Grubbs EG, Ingledew P-A. An analysis of the quality of thyroid cancer websites.Endocrine Practice, 2019; 25(10): 1003-1011.

Cnossen IG, van Uden-Kraan CF, Eerenstein SEJ, Rinkel RNPM, Aalders IJ, van den Berg K, *et al*. A Participatory Design Approach to Develop a Web-Based Self-Care Program Supporting EarlyRehabilitation among Patients after Total Laryngectomy. Folia Phoniatrica et Logopaedica 2015; 67:193–201.

Cochrane Database of Systematic Reviews [Internet]. Global [cited February 19 2021]. Availablefrom:

https://cochranelibrary.com

Duman-Lubberding S, van Uden-Kraan CF, Peek N, Cuijpers P, Leemans CR, Verdonck-de-LeeuwIM. An eHealth Application in Head and Neck Cancer Survivorship Care: Health Care Professionals' Perspectives. Journal of Medical Internet Research 2015; 17(10): e235.

Fallon EA, Driscoll D, Smith TS, Richardson K, Portier K. Description, characterization, andevaluation of an online social networking community: the American Cancer Society’s CancerSurvivors Network® Journal of Cancer Survivorship 2018; 12:691–701.

Fitzmaurice C. Global, Regional, and National Cancer Incidence, Mortality, Years of Life Lost, Years Lived With Disability, and Disability- Adjusted Life-years for 32 Cancer Groups, 1990 to 2015 A Systematic Analysis for the Global Burden of Disease Study. JAMA Oncology 2017; 3(4),524-548.

Hakim, M., Billan, S., Tisch, U., Peng, G., Dvrokind, I., Marom, O., Abdah-Bortnyak, R., Kuten,A., Haick, H. Diagnosis of head-and-neck cancer from exhaled breath. British Journal of Cancer 2011; 104, 1649 – 1655.

Hinni, ML., Ferlito, A., Brandwein-Gensler, MS., Takes, RP., Silver, CE., Westra, WH., Seethala,RR., Rodrigo, JP., Corry, J., Bradford, CR., Hunt, JL., Strojan, P., Devaney, KO., Gnepp, DR., Hartl, DM., Kowalski, PL., Rinaldo, A., Barnes, L. Surgical margins in head and neck cancer: A contemporary review. Head and Neck 2012; 35(9): 1362- 1670.

Hoogland AI, Mansfield J, Lafranchise EA, Bulls HW, Johnstone PA, Jim HSL. eHealth literacy inolder adults with cancer. Journal of Geriatric Oncology 2020; 11: 1020–1022.

Köhle N, Drossaert CHC, Van Uden-Kraan CF, Schreurs KMG, Hagedoorn M, Verdonck-de Leeuw IM, Bohlmeijer ET. Intent to use a web-based psychological intervention for partners of cancer patients: Associated factors and preferences. Journal of Psychosocial Oncology 2018; 36:2,203-221.

Malik TAM, Heywood EG, O’Connor TJ, Baker DM, Marshall JH, Beasley N. YouTube™ as asource of information for patients undergoing laryngectomy: a thematic analysis. European Archives of Oto-Rhino-Laryngology 2019; 276: 3213–3219.

Manne S, Hudson S, Frederick S, Mitarotondo A, Baredes S, Kalyoussef E, Ohman-Strickland P,Kashy DA. e-Health self-management intervention for oral and oropharyngeal cancer survivors: design and single-arm pilot study of empowered survivor. Head & Neck 2020 in press; 1–14.

Pagedar NA, Schularick NM, Lee PC, Karnell LH. Health-Related Internet Use Among Otolaryngology Patients. Annals of Otology, Rhinology & Laryngology 2018; 127(8): 551–557.

Parahoo RS, Semple CJ, Killough S, McCaughan E. The experience among patients with multipledental loss as a consequence of treatment for head and neck cancer: A qualitative study. Journal ofDentistry 2019; 82: 30-37.

Peters MDJ, Godfrey C, McInerney P, Baldini Soares C, Khalil H, Parker D. Chapter 11: ScopingReviews. In: Aromataris E, Munn Z (Editors). Joanna Briggs Institute Reviewer’s Manual.
Adelaide: Joanna Briggs Institute; 2017. [internet]. [cited January 25 2021]. Available from: https://reviewersmanual.-joannabriggs.org/.

Peterson SK, Shinn EH, Basen-Engquist K, Demark-Wahnefried W, Prokhorov AV, Baru C, *et al.*Identifying Early Dehydration Risk With Home-Based Sensors During Radiation Treatment: A Feasibility Study on Patients With Head and Neck Cancer. Journal of National Cancer Institute Monographs 2013; 47:162–168.

Saroa O, Molzahn AE, Northcott HC, Schmidt K, Ghosh S, Olso, K. A Survey of Information Needs and Preferences of Patients With Head and Neck Cancer. Oncology Nursing Forum 2018;45(6): 761–774.

Schwarzbach HL, Mady .J, Kaffenberger TM, Duvvuri U, Jabbour N. Quality and Readability Assessment of Websites on Human Papillomavirus and Oropharyngeal Cancer. Laryngoscope 2020in press; 1–8.

Seale B. Patients as partners: Building collaborative relationships among professionals, patients, carers and communities. [Internet]. The King's Fund .2016 [cited 26 Mar 2019]. Available from https://www.kingsfund.org.uk/sites/default/files/field/field_publication_file/Patients_as_partners.pd f

Semple CJ, Killough S. Quality of life issues in head and neck cancer. Dental Update 2014; 41 (4):346-353.

Semple CJ, Rutherford H, Killough S, Moore C, McKenna G. Long-term impact of living with anobturator following a maxillectomy: A qualitative study. Journal of Dentistry 2019; 90: 103212.

Shinn, E.H., Jensen, K., McLaughlin, J., Garden, A.S., Fellman, B.M., Liang, L. & Peterson, S.K.Interactive website for head and neck cancer patients: Adherence and coping program to prevent dysphagia after radiation. Internet Interventions 2019; 18: 100289.

Sosa A, Heineman N, Thomas K, Tang K, Feinstein M, Martin MY, *et al*. Improving Patient HealthEngagement with Mobile Texting: A Pilot Study in the H&N Post-Operative Setting. Head Neck 2017; 39(5): 988–995.

Tricco AC, Lillie E, Zarin W, O'Brien KK, Colquhoun H, Levac D, *et al*. PRISMA Extension for Scoping Reviews (PRISMA-ScR): Checklist and Explanation. Annals of Internal Medicine 2018;169: 467-473.

Van der Hout A, van Uden-Kraan CF, Holtmaat K, Jansen F, Lissenberg-Witte BI, Nieuwenhuijzen GAP, *et al*. Role of eHealth application Oncokompas in supporting self- management of symptoms and health-related quality of life in cancer survivors: a randomised,controlled trial. Lancet Oncology 2019; 21: 80–94

Appendix I: Search strategies

	CINAHL	MEDLINE Ovid	SCOPUS	EMBASE	WoS	COCHRANE	PUBMED	PsychInfo
Studies<br>S1 Web-based interventions AND headand neck cancer								
S2 Web based interventions AND headand neck cancer AND diagnosis & treatment								
S3 Online resources AND head and neck cancer								
Websites<br>Head and neck cancer	**GOOGLE**							

Appendix II: Data extraction instrument for included papers

Author/Year	Title	Journal	Database	Search terms	Design	Outcomes	Final inclusion

Appendix III: Data presentation template

Citation	Country of origin	Study Population	Concept	Context	Design	Key finding related to researchquestion

Online resource 2 Data Extraction Table for empirical studies

Article title: Availability and use of web-based resource for patients with head and neck cancer: a scoping review

Journal: Journal of Cancer Survivorship

Authors: Kelly R, , Semple CJS, .Gordon P, & Thompson R.

Corresponding author: Semple CJS, cherith.semple@setrust.hscni.net

Author/Year	Title	Journal	Database	Search terms	Design	Outcomes	Final inclusion
Alsoghier et al (2018)	Web-based information on oral dysplasia and precancer of the mouth –Quality and readability	Oral Oncology	PubMed	Online resources + HNC	The first 100 sites identified from 9 searches were assessed for quality, content and readability using validated tools.	There was little reliable information available for patients on oral epithelial dysplasia.	Exclude. Not related to HNC patients.
Badr et al (2016)	Development and usability testing of a web-based self-management intervention for oral cancer survivors and their family caregivers	European Journal of Cancer Care	Medline Ovid	WBI + HNC	Development and evaluation of a web-based intervention (CARES) using Grounded Theory	Five educational models were developed which survivors and carers tested for usability. Tailored information for patients and carers of most benefit.	Include
Bakitas et al (2017)	"There were more decisions and more options than just yes or no": Evaluating a decision aid for advanced cancer patients and their	Palliative & Supportive Care	CINAHL Complete	WBI + HNC	Evaluation of a specific element of a patient-decision aid exploring advance	Evidence of tool helping patients understand and discuss future healthcare decision-making.	Exclude. No evidence of head and neck cancer patients being involved in the

Author/Year	Title	Journal	Database	Search terms	Design	Outcomes	Final inclusion
	family caregivers				care planning.		study.
Biggs et al (2018)	Quality of online otolaryngology health information	Journal of Laryngology & Otology	Scopus	Online resources + HNC	Independent analysis of websites by two clinicians	The 2 cancer sites were rated highly for their information on a range of conditions. Wikipedia was rated lower but contained mostly accurate information.	Include
Bond et al (2020)	Initial psychometric testing of the Head and Neck Cancer Patient Self-Management Inventory (HNC-PSMI)	European Journal of Oncology Nursing	Scopus	WBI + HNC	A cross-sectional survey	The domains provided evidence of the high burden of self-management, resulting in gaps in care when patients were unable to manage.	Exclude - methodology.
Brady et al (2020)	Using experience-based co-design to improve the pre-treatment care pathway for people diagnosed with head and neck cancer	Supportive Care in Cancer	PubMed	Online resources + HNC	An experience-based co-design approach	Patients highlighted the need for different information methods including online/digital resources.	Include
Chang et al (2019)	An analysis of the quality of thyroid cancer websites	Endocrine Practice	PubMed	Online resources + HNC	A validated structured rating tool was used to evaluate online thyroid cancer patient information quality.	Only 2% of websites were readable without high school education. 18% required education beyond high school. Definition, treatment and diagnosis were the most common topics covered, sometimes incompletely or inaccurately.	Include
Cnossen et al (2015)	A Participatory Design Approach to Develop a Web-Based Self-Care Program Supporting Early Rehabilitation among Patients after Total Laryngectomy	Folia Phoniatrica et Logopaedica	Scopus	WBI + HNC	Focus groups, usability testing and interviews.	A number of strengths and weakness with the platform were identified. A refined prototype will be developed for a further feasibility study.	Include
Duman-Lubberding et al (2015)	An eHealth Application in Head and Neck Cancer Survivorship Care: Health Care Professionals'	Journal of Medical Internet Research	WoS	WBI + HNC	HCP interviews and cognitive walkthroughs	Several points were identified for optimising the prototype and developing an efficient	Include

Author/Year	Title	Journal	Database	Search terms	Design	Outcomes	Final inclusion
	Perspectives					implementation strategy.	
Elwyn et al (2011)	How to develop web-based decision support interventions for patients: A process map	Patient Education and Counselling	CINAL Complete	WBI + HNC	Co-production	Patient perspective is a key element of content specification. An interactive method of trial and adaptation should be used during the development process.	Exclude. Methodology only.
Fallon et al (2018)	Description, characterization, and evaluation of an online social networking community: the American Cancer Society's Cancer Survivors Network®	Journal of Cancer Survivorship	Scopus	Online resources + HNC	Online self-report survey and digital metrics of site use.	Such sites may be beneficial for support at multiple points during cancer experiences.	Include
Gharzai et al (2020)	Perspectives on Oncology-Specific Language During the Coronavirus Disease 2019 Pandemic A Qualitative Study	JAMA Oncology	PubMed	Online resources + HNC	Semi-structured interview by email or telephone with staff. Anonymous online survey for patients.	The emotional patient responses informed the selection of the 3 general communication principles, which suggested language and strategies that physicians can use to respond to patients.	Exclude – not relevant to study aim.
Golită and Băban (2019)	A systematic review of the effects of internet-based psychological interventions on emotional distress and quality of life in adult cancer patients	Journal of Psychosomatic Research	WoS	WBI + HNC	Systematic review	Web-based delivery format show potential in effective management of psychological distress and QOL in cancer patients	Exclude. Evidence synthesis
Goode et al (2015)	Telephone, print, and Web-based interventions for physical activity, diet, and weight control among cancer survivors: a systematic review	Journal of Cancer Survivorship	WoS	WBI + HNC	Systematic review	Broad-reach intervention delivery modalities for healthy lifestyle programs provide a means to meet the needs of a growing and disparate group of cancer survivors and have strong potential to improve health behaviors and in turn treatment-related side effects, quality of life, and health	Exclude. No reference to papers relating to HNC.

Author/Year	Title	Journal	Database	Search terms	Design	Outcomes	Final inclusion
						outcomes.	
Greenhalgh & Russell (2010)	Why Do Evaluations of eHealth Programs Fail? An Alternative Set of Guiding Principles	PLoS Medicine	CINAL Complete	WBI + HNC	Discussion paper	Different approaches may be used in evaluation- scientific or alternative. A debate on ways of knowing in eHealth evaluation is needed.	Exclude. Methodology only.
Groen et al (2015)	Empowerment of Cancer Survivors Through Information Technology: An Integrative Review	Journal of Medical Internet Research	PsychInfo	WBI + HNC	Integrative review	5 main attributes of patient empowerment and 5 main categories of IT services were identified.	Exclude. Evidence syntheisis
Hoogland et al (2020)	eHealth literacy in older adults with cancer	Journal of Geriatric Oncology	Scopus	Online resources + HNC	Anonymous survey	Older participants were less likely to feel confident in evaluating internet health resources and how to use them.	Include
Kaltenbaugh et al (2015)	Using Web-Based Interventions to Support Caregivers of Patients With Cancer: A Systematic Review	Oncology Nursing Forum	CINAHL Complete	WBI + HNC	Systematic review	The authors claim that this body of work supports the adaptation of cancer caregiver interventions to a web-based format.	Exclude. Evidence synthesis
Kim and Park (2015)	Web-based Self-management Support Interventions for Cancer Survivors: A Systematic Review and Meta-analyses	Studies in Health Technology and Informatics	CINAHL Complete	WBI + HNC	Systematic review	The most popular mode of intervention delivery was “peer-to-peer access." The effects across all outcome measures were small to moderate compared to standard care.	Exclude. No evidence of HNC in characteristics list.
Köhle et al (2017)	User-experiences with a web-based self-help intervention for partners of cancer patients based on acceptance and commitment therapy and self-compassion: a qualitative study	BMC Public Health	WoS	WBI + HNC	Individual in-depth interviews with partners of cancer patients who used the Web-based self-help intervention.	Partners appreciated the intervention, however, they also expressed ambivalent feelings towards peer support, the content of the feedback of their counselor, and the ‘tunneled’ structure of the intervention.	Exclude. No involvement of HNC patients.

Author/Year	Title	Journal	Database	Search terms	Design	Outcomes	Final inclusion
Köhle et al (2018)	Intent to use a web-based psychological intervention for partners of cancer patients: Associated factors and preferences	Journal of Psychosocial Oncology	PubMed	WBI + HNC	A web-based and paper survey	Only 13% of participants were interested in such an intervention. The intention to use a psychological web-based intervention was not associated with levels of psychological distress.	Include
Lleras de Frutos et al (2020)	A systematic and comprehensive review of internet use in cancer patients: Psychological factors	Psycho-Oncology	Scopus	Online resources + HNC	Systematic review	Results described emotional and behavioral outcomes in cancer patients who accessed online information.	Exclude – evidence synthesis.
Malik et al (2019)	YouTube™ as a source of information for patients undergoing laryngectomy: a thematic analysis	European Archives of Oto-Rhino-Laryngology	WoS	Online resources + HNC	Assessment of the thematic content of the most viewed YouTube videos concerning laryngectomy and to evaluate user response	3 themes and 17 subthemes were identified. There is limited evidence of the quality of this information.	Include
Manne et al (2020)	e-Health self-management intervention for oral and oropharyngeal cancer survivors: design and single-arm pilot study of empowered survivor	Head & Neck	WoS	WBI + HNC	Single-arm study with a pre-, 2-month, and 6-month post intervention survey.	Participants felt the tool was easy to navigate and presented valid and useful information, and they felt the modules were helpful in navigating survivorship self-care. A larger RCT is needed.	Include
Nguyen-Feng et al (2020)	Web-Based Perceived Present Control Intervention for Voice Disorders: A Pilot Study	Journal of Voice	Scopus	WBI + HNC	A pilot study of a web-based intervention for patients with voice disorders.	The study found high completion rates and most participants planned to continue the control exercises after study completion.	Exclude. None of the patients had cancer.
Pagedar et al (2018)	Health-Related Internet Use Among Otolaryngology Patients	Annals of Otology, Rhinology and	Embase	Online resources + HNC	Survey	A third of patients used the Internet to research their health condition prior to their appointment.	Include

Author/Year	Title	Journal	Database	Search terms	Design	Outcomes	Final inclusion
		Laryngology					
Peterson et al (2013)	Identifying Early Dehydration Risk With Home-Based Sensors During Radiation Treatment: A Feasibility Study on Patients With Head and Neck Cancer	Journal of the National Cancer Institute Monographs	Scopus	WBI + HNC	Feasibility study.	The results showed both feasibility and high acceptability among patients and professionals. An RCT is recommended.	Include
Pfirrmann et al (2017)	Internet-based perioperative exercise program in patients with Barrett's carcinoma scheduled for esophagectomy [iPEP - study] a prospective randomized controlled trial	BMC Cancer	PubMed	WBI + HNC	Prospective multi - centre RCT	Study protocol only	Exclude. No patient data available - protocol.
Prabhu et al (2016)	Radiation Oncology and Online Patient Education Materials: Deviating From NIH and AMA Recommendations	International Journal of Radiation Oncology Biology Physics	PubMed	Online resources + HNC	10 quantitative readability scales were used to assess the readability level of online patient education resources for radiation oncology.	Only 1 article met the US recommendation that patient education resources should be written between 3rd-7th grade levels.	Exclude – no
Roe et al (2012)	Assessment and management of dysphagia in patients with head and neck cancer who receive radiotherapy in the United Kingdom – A web-based survey	Oral Oncology	WoS	WBI + HNC	Service evaluation	Survey responses demonstrated considerable variability in service provision.	Exclude. No patient involvement
Saroa et al (2018)	A Survey of Information Needs and Preferences of Patients With Head and Neck Cancer	Oncology Nursing Forum	Scopus	Online resources + HNC	Self-administered survey	Patients wanted information on signs of recurrence; cure rates; post treatment rehab; treatment and recovery time; financial assistance.	Include

Author/Year	Title	Journal	Database	Search terms	Design	Outcomes	Final inclusion
Schwarzbach et al (2021)	Quality and Readability Assessment of Websites on Human Papillomavirus and Oropharyngeal Cancer	The Laryngoscope	PubMed	Online resources + HNC	Cross-sectional website analysis.	The currently available online information about HPV+ oropharyngeal cancer was found to be insufficient.	Include
Semple et al (2013)	Psychosocial interventions for patients with head and neck cancer	Cochrane Library	Cochrane Database of Systematic Reviews	WBI + HNC	Review of trials of psychosocial interventions	Evidence was limited with several methodological shortcomings.	Exclude as evidence synthesis.
Shim et al (2011)	Comprehensive needs assessment tool in cancer (CNAT): the development and validation	Supportive Care in Cancer	CINAHL Complete	WBI + HNC	Development and evaluation of a needs assessment tool for cancer (CNAT).	Tool reported as enabling assessment of need in cancer care. No specific inclusion of head and neck cancer patients.	Exclude. No specific inclusion of head and neck cancer patients.
Shinn et al (2019)	Interactive website for head and neck cancer patients: Adherence and coping program to prevent dysphagia after radiation.	Internet Interventions	Scopus	WBI + HNC	Interim study analysis of a 10-week web-based intervention	At the time of reporting all had baseline data collected. the study has demonstrated improvement in adherence to swallowing exercises, it has also demonstrated usability improvements needed for the platform to meet the needs of an older population.	Include.
Sosa et al (2017)	Improving Patient Health Engagement with Mobile Texting: A Pilot Study in the H&N Post-Operative Setting	Head Neck	Medline Ovid	WBI + HNC	Prospective pilot of an automated text-based intervention	Results suggest that the use of a text message platform in the acute post-operative setting is feasible and potentially improves head and neck cancer patient engagement with treatment.	Include
Van er Hout et al (2019)	Role of eHealth application Oncokompas in supporting self-management of symptoms and health-related quality of life in cancer survivors: a randomised, controlled trial	Lancet Oncology	Medline Ovid	WBI + HNC	Non-blinded RCT.	The results revealed no improvement in the amount of knowledge, skills and confidence for self-management in cancer survivors.	Include

Author/Year	Title	Journal	Database	Search terms	Design	Outcomes	Final inclusion
Yildirim et al (2019)	An Evaluation of the Information Sources of Cancer Patients' Relatives. A Prospective Survey	Journal of Cancer Education	CINAHL Complete	WBI + HNC	Questionnaire	Oncologists were the primary information source followed by the internet.	Exclude. No evidence of carers of head and neck patients being involved in the study.

Online resource 2 Data extraction table for websites

Link	Country	Content	Final inclusion
www.nhs.uk/conditions/head-and-neck-cancer	UK	Information on symptoms, causes, diagnosis, treatment, living with, and complications	Yes
www.macmillan.org.uk/cancer-information	UK	Information and support	Yes
www.cancerresearchuk.org	UK	Information and support	Yes
www.cancer.gov/types/head-and-neck	USA	Information and support	Yes
www.wikipedia.org/wiki/Head_and_neck_cancer	International	Information on diagnosis & treatment	Yes
www.wikidoc.org/index.php/Head_and_neck_cancer	International	Diagnosis	Yes
www.nice.org.uk	UK	Guidance (27) Advice (1) pathways (6) local practice (2) news (6) quality standards (1)	Yes
www.medicaldictionary.thefreedictionary.com	International	Only provides definitions	No
www.guysandstthomas.nhs.uk/our-services/cancer	UK	Information on services, treatment and support	No
www.mayoclinic.org/diseases-conditions/head-and-neck-cancers	USA	Information on services and sub types	No
www.hncf.org.uk	UK	Diagnosis and treatment	No

Link	Country	Content	Final inclusion
www.cdc.gov/cancer/headneck	USA	Diagnosis; prevention; links to other sites	Yes
www.cancer.net	USA	Information and support	Yes
www.ouh.nhs.uk/cancer/cancer-by-type/head-and-neck	UK	Details of service personnel and services. Links to other sites for information and support. Large selection of patient information leaflets	Yes
www.scan.scot.nhs.uk/CancerInformation/headneck	UK	Symptoms, diagnosis, treatment, patient information leaflets	No
www.hancuk.org	UK	Patient stories; information and support	Yes
www.christie.nhs.uk	UK	Information and support; services, PALS	Yes
www.radiologyinfo.org	USA	Radiation information for patients. Sub section provides information on various options for radiation therapy in HNC	Yes
www.royalmarsden.nhs.uk	UK	Details of services and information and support. Dedicated head and neck page.	Yes
www.headandneck.org	USA	Survivorship webinars/videos; information and support	Yes
www.nuffieldhealth.com	UK	Links from main page to treatments and services available	No
www.bahno.org.uk	UK	Professional organisation with patient area. Contains info on prevention and symptoms	Yes
www.ncin.org.uk	UK	For professionals but contains patient/public page with links to other organisations such as Macmillan, along with cancer terms and glossary	No
www.plymouthhospitals.nhs.uk	UK	Can navigate from home page to HNC page. Information about services, treatment and support	No
www.bccancer.bc.ca	Canada	Links to a number of guides for patients attending the centre. Dedicated page for HNC with information and support links	Yes
www.gloshospitals.nhs.uk	UK	Information on services available in the hospital. Lnks to other webpages for further support and information.	No
www.cancerresearch.org	UK	Dedicated to immunotherapy. Lots of examples of patient stories	No
www.stgeorges.nhs.uk/had-and-neck-cancer	UK	Info on services with links to support organisations such as Macmillan.	No

Link	Country	Content	Final inclusion
www.merckgroup.com	Germany	Company webpage available to the public. Several patient experience videos and infographics on site	Yes
www.ucl.ac.uk/cancer/research/department	UK	Details of research currently being performed. Includes page on HNC research study	No
www.cancercare.org/diagnosis/head-and-neck-cancer	USA	Information on support and information including podcasts, workshops and publications. Board for questions to be submitted and general topic info. Links to other organisations and webpages	Yes
www.cancercouncil.com.au/head-and-neck-cancer	Australia	Info on subtypes (very good illustrations) Links to other support and information	Yes
www.kch.nhs.uk/cancer/cancer-types/head-and-neck	UK	Direct link to cancer info from home page. Includes info on HNC, services, treatment and support.	No
www.nottshncs.nhs.uk/head-and-neck-cancer-patients	UK	Treatment options, care and support for HNC patients.	No
www.utswmed.org	USA	Information on services, subtypes, treatment and support.	No
www.asha.org/Head-and-Neck-Cancer	USA	Information on subtypes, treatment , evidence map, support, resources.	No
www.moffitt.org/cancers/head-and-neck-cancer/faqs	USA	Information on diagnosis, treatment, FAQs. Patient experience videos	Yes
www.cancertherapyadvisor.com/home/cancer-topics	USA	Research and treatment news, treatment regimes	No
www.cancervic.org.au	Australia	Information on overview, diagnosis, treatment	Yes
www.webmd.com	USA	Series of links to HNC diagnosis, treatment options	No
www.bapras.org.uk/head-and-neck-cancer	UK	Information on plastic surgery options following HNC. Information and illustrations on HNC. Link to Macmillan support	No
www.cancerquest.org/patients/cancer-type	USA	Link to HNC webpage for patients. Developed in consultation with patients. Information on anatomy, types, risk factors, symptoms, diagnosis, staging, treatment, survivorship, international resources (links to services across the world)	Yes
www.mskcc.org/cancer-care/types/head-neck	USA	Information on signs and symptoms, diagnosis, treatment, survivorship	Yes
www.leicesterhospitals.nhs.uk/aboutus	UK	Information on services, diagnosis, treatment and support groups.	No

Link	Country	Content	Final inclusion
www.cancer.ucla.edu/information-for-patients	USA	Information on signs and symptoms, diagnosis, treatment and services.	No
www.aacr.org/patients-caregivers/cancer/head-and-neck	USA	Information on subtypes, survivorship videos and patient advocacy section	Yes
www.mylifehouse.org.au/for-patients/cancer-types	Australia	HNC page covers diagnosis, treatment, support. Tab for patients/caregivers. Includes links to support groups and beyondfive page	Yes
www.cancer.org.au	Australia	Sections on Talk to Someone (Support groups, telephone peer support), Cultural Resources, Practical Assistance, Emotional Assistance, Online support services including e-learning	Yes
www.cancer.ie/mouth-head-and-neck-cancer	Ireland	Information on diagnosis, treatment, support and publications	Yes
www.uhb.nhs/uk/Downloads/pdf/PiMaxillofacial/Diet	UK	Information leaflet on diet and drinks following surgery.	No
www.albertahealthservices.ca	Canada	Link to a resources pages with links to other cancer services nationally and internationally	No
www.wiki.cancer.org.au/Australia/COSA:Head_and_neck	Australia	Clinical guidelines including one for Barrett's Oesophgus and HNC nutrition	No
www.foxchase.org	USA	Patient and families support services section . Page on HNC including diagnosis, treatment, survivorship, patient stories and clinical trials	Yes
www.alaskaradiationtherapy.com/anchorage	USA	Information on radiation treatments available locally, support service locally and nationally	No
www.curetoday.com/articles	USA	Provides links to news items and resources related to HNC	No
www.uhn.ca/Patients/Families/Health_Information	Canada	Links to patient resources available at cancer centre including information leaflets and videos on treatments and survivorship	No
www.networks.nhs.uk/nhs-networks/dental-lpn	UK	Provides 3 pages of results relating to HNC including HNC and sexuality, audits, restorative dental care.	No
www.fredhutch.org/en/news/center-news/2014/08	USA	Research facility. HNC page list results relating to HNC research and guideline development	No
www.cancertodaymag.org/Pages/cancer-talk/Coping	USA	Several tabs on home page - types of cancer, research, living with cancer, support, taking action (advocacy, survivorship), cancer talk	No

Link	Country	Content	Final inclusion
		(blog).	
www.ahns.info/resources/education/patient_education	USA	Tab For Patients includes - survivorship, interviews, HPV vaccine for boys, Find a physician	Yes
www.enthealth.org/conditions	USA	HNC page covers symptoms, treatment, questions for doctors	Yes
www.headandneckcancer.org.au	Australia	Website dedicated to HNC for patients/families/professionals.	Yes

Online resource 3 QUEST Criteria

Article title: Availability and use of web-based interventions for patients with head and neck cancer: A Scoping Review

Journal: Journal of Cancer Survivorship

Authors: Kelly R, Semple CJS, Gordon P, & Thompson R.

Corresponding author: Semple CJS, cherith.semple@setrust.hscni.net

Authorship **(Score x 1)**
0 – No indication of authorship or username
1 – All other indications of authorship
2 – Author's name and qualification clearly stated

Attribution **(Score x 3)**
0 – No sources
1 – Mention of expert source, research findings (though with insufficient information to identify the specific studies), links to various sites, advocacy body, or other
2 – Reference to at least one identifiable scientific study, regardless of format (e.g., information in text, reference list)
3 – Reference to mainly identifiable scientific studies, regardless of format (in >50 of claims)

For all articles scoring 2 or 3 on Attribution: **(Score x 1)**
Type of study

0 – In vitro, animal models, or editorials
1 – All observational work
2 – Meta-analyses, randomized controlled trials, clinical studies

Conflict of interest **(Score x 3)**
0 – Endorsement or promotion of intervention designed to prevent or treat condition (e.g., supplements, brain training games, foods) within the article
1 – Endorsement or promotion of educational products & services (e.g., books, care home services)
2 – Unbiased information

Currency **(Score x 1)**
0 – No date present
1 – Article is dated but 5 years or older
2 – Article is dated within the last 5 years

Complementarity **(Score x 1)**
0 – No support of the patient-physician relationship
1 – Support of the patient-physician relationship

Tone (includes title) **(Score x 3)**
0 – Fully supported (authors fully and unequivocally support the claims, strong vocabulary such as "cure", "guarantee", and "easy", mostly use of non-conditional verb tenses ("can", "will"), no discussion of limitations)
1 – Mainly supported (authors mainly support their claims but with more cautious vocabulary such as "can reduce your risk" or "may help prevent", no discussion of limitations)
2 – Balanced/cautious support (authors' claims are balanced by caution, includes statements of limitations and/or contrasting findings)
